# Socioeconomic Inequalities Increase the Probability of Ketoacidosis at Diagnosis of Type 1 Diabetes: A 2014–2016 Nationwide Study of 2,679 Italian Children

**DOI:** 10.3389/fped.2020.575020

**Published:** 2020-10-22

**Authors:** Rosaria Gesuita, Claudio Maffeis, Riccardo Bonfanti, Francesca Cardella, Felice Citriniti, Giuseppe D'Annunzio, Adriana Franzese, Dario Iafusco, Antonio Iannilli, Fortunato Lombardo, Giulio Maltoni, Ippolita Patrizia Patera, Elvira Piccinno, Barbara Predieri, Ivana Rabbone, Carlo Ripoli, Sonia Toni, Riccardo Schiaffini, Renee Bowers, Valentino Cherubini, Network of the Italian Society of Pediatric Endocrinology and Diabetes (ISPED) for DKA Study and Prevention

**Affiliations:** ^1^Centre of Epidemiology and Biostatistics, Polytechnic University of Marche, Ancona, Italy; ^2^Pediatric Diabetes and Metabolic Disorders Unit, University of Verona School of Medicine and Surgery, Verona, Italy; ^3^Department of Pediatrics, San Raffaele Institute, Milan, Italy; ^4^Division of Pediatrics, Ospedale dei Bambini, Palermo, Italy; ^5^Azienda Ospedaliera Pugliese Ciaccio, Catanzaro, Italy; ^6^Pediatric Clinic and Endocrinology, Regional Reference Center for Pediatric Diabetes, IRCCS Istituto Giannina Gaslini, Genoa, Italy; ^7^Department of Translational Medical Science, Section of Pediatrics, University of Naples Federico II School of Medicine and Surgery, Naples, Italy; ^8^Department of Pediatrics, Regional Center of Pediatric Diabetology “G. Stoppoloni”, University of Campania “Luigi Vanvitelli”, Naples, Italy; ^9^Department of Women's and Children's Health, Azienda Ospedaliero Universitaria Ospedali Riuniti di Ancona Umberto I G M Lancisi G Salesi, Ancona, Italy; ^10^Department of Human Pathology in Adult and Developmental Age “Gaetano Barresi”, University of Messina, Messina, Italy; ^11^Department of Pediatrics, University Hospital of Bologna Sant'Orsola-Malpighi Polyclinic, Bologna, Italy; ^12^Ospedale Pediatrico Bambino Gesù, Endocrinology and Diabetes Unit, Roma, Italy; ^13^Unitá Operativa Complessa (UOC) Malattie Metaboliche e Diabetologia, Ospedale Pediatrico Giovanni XXIII, Bari, Italy; ^14^Pediatric Unit, Department of Medical and Surgical Sciences of the Mother, Children and Adults, University of Modena and Reggio Emilia, Modena, Italy; ^15^Division of Pediatrics, Department of Health Sciences, University of Piemonte Orientale, Vercelli, Italy; ^16^Department of Pediatrics, Azienda Ospedaliera G. Brotzu Cagliari, Cagliari, Italy; ^17^Meyer Children's Hospital, Pediatric Endocrinology and Diabetology Unit, Firenze, Italy; ^18^Population Health, Faculty of Health Sciences, University of Ottawa, Ottawa, ON, Canada

**Keywords:** DKA (diabetic ketoacidosis), type 1 diabetes (T1D), socioeconomic factors, inequalities, children

## Abstract

This study aims to compare the frequency of Diabetic Ketoacidosis (DKA) at diagnosis in 2014–2016 with the one previously reported in 2004–2013; and to assess the association between family socioeconomic status and DKA at type 1 diabetes (T1D) diagnosis in children <15 years of age from 2014 to 2016.

**Methods:** This nationwide, population-based, observational study included 2,679 children diagnosed with T1D from 54 Italian centers for pediatric diabetes during 2014–2016. The ISPAD criteria for DKA were used as a standard reference. The overall and by age frequency of DKA between the two time periods were compared. The association between family socioeconomic status and DKA was assessed using multiple logistic regression analysis.

**Results:** Nine hundred and eighty nine children had DKA (36.9, 95% CI: 35.1–38.8). The frequency of DKA was significantly lower in 2014–2016 in comparison to 2004–2013 (40.3, 95% CI: 39.3–41.4, *p* = 0.002). The probability of having DKA at diagnosis was lower in mothers with a high level of education (OR = 0.69, 95% CI: 0.51–0.93) or a high level of occupation (OR = 0.76, 95% CI: 0.58 0.99), and in fathers with a high level of occupation (OR = 0.72, 95% CI: 0.55–0.94). Children living in Southern Italy had a higher probability of diagnosis with severe DKA than children living in Central Italy.

**Conclusion:** There was a decrease in the frequency of DKA in children diagnosed with T1D under 15 years of age during 2014–2016. However, DKA frequency remains unacceptably high. This study demonstrated that socioeconomic inequalities, measured as low education and occupational levels, were associated with an increased probability of DKA at T1D diagnosis.

## Introduction

Diabetic ketoacidosis (DKA) is a life-threatening complication that frequently affects children and adolescents with newly diagnosed type 1 diabetes (T1D). This condition is associated with an increase in morbidity and mortality, poor long-term metabolic control (1), changes in brain imaging and low cognitive test scores in young children after a single episode of moderate/severe DKA (2), and high health costs (3). The presence of DKA at the onset of diabetes may be a consequence of a delay in referral of children with suspected T1D (4) or income inequality (5).

In Italy, the frequency of DKA at T1D diagnosis was astonishingly high (40.3, 95% CI: 39.3–41.4), during 2004–2013 (6). Moreover, a 2020 study analyzed temporal trend of DKA at T1D diagnosis during 2006–2016 in 13 countries spanning three continents. The study reported an overall slight increase and a huge geographical variation of DKA prevalence; while in Italy the prevalence was very high (41.2, 95% CI: 40.3–42.2), a slight decrease was observed over the study time period (7). These two studies show that the frequency of DKA at T1D diagnosis remains high in Italy. This finding is surprising since the National Health Service in Italy provides healthcare, medications, and diabetes management devices free of charge to all European Union residents and non-European Union citizens holding a residence permit who live in Italy, irrespective of income, gender or other factors. Moreover, health interventions were recently implemented to promote awareness of symptoms of diabetes amongst the general public and health care professionals.

The published literature over the last 20 years suggests that awareness campaigns for both the public and health professionals are an effective method to improve awareness of initial symptoms of T1D and encourage an early diagnosis (8). Different campaigns have been used in different settings. These ranged from the simplest of tools such as posters, postcards, explanatory letters, and leaflets to the general public, to more complex tools such as periodic reminders for healthcare professionals, and teachers on the risk of developing DKA due to delayed diagnosis (9).

Based on this literature, the Italian Society for Pediatric Endocrinology and Diabetology (ISPED) started a national awareness campaign for both the public and health professionals in 2014 (10). This campaign remains active and includes advertising posters and social media reminders (Facebook, Instagram, and advertisements on local television channels) for the public. For health professionals, recommendations were published (11) and disseminated to pediatricians and diabetologists for the management of DKA. However, a recent systematic review concluded that variations in awareness campaign implementation, and subsequent evaluation, make it difficult to determine their effectiveness (8). This recent finding, and the current implementation stage of the campaign in Italy, prompted consideration of factors beyond awareness that affect the probability of DKA at diagnosis of T1D.

One of these considerations is socioeconomic status (SES). To date, few studies have analyzed the association between SES and DKA at T1D diagnosis. Factors examined in available studies were family income indicators, insurance and ethnicity (12, 13). A study on the impact of these factors is a gap in the literature for children in Italy with T1D.

This study compared the reported frequency of DKA at diagnosis for two time periods, 2014–2016 and a 10 year period, 2004–2013. Moreover, we hypothesized that parents' lower socioeconomic status is associated with a higher risk of having DKA at T1D diagnosis in children under 15 years in Italy.

## Methods

The Network of ISPED for DKA Study and Prevention, which includes all the Italian centers (*n* = 58) for diabetes in children and adolescents, repeated a nationwide longitudinal population-based study on DKA at diagnosis of T1D in 2014–2016. The same data collection methods were used (6) in both studies.

### Data Source

**Figure 1:**
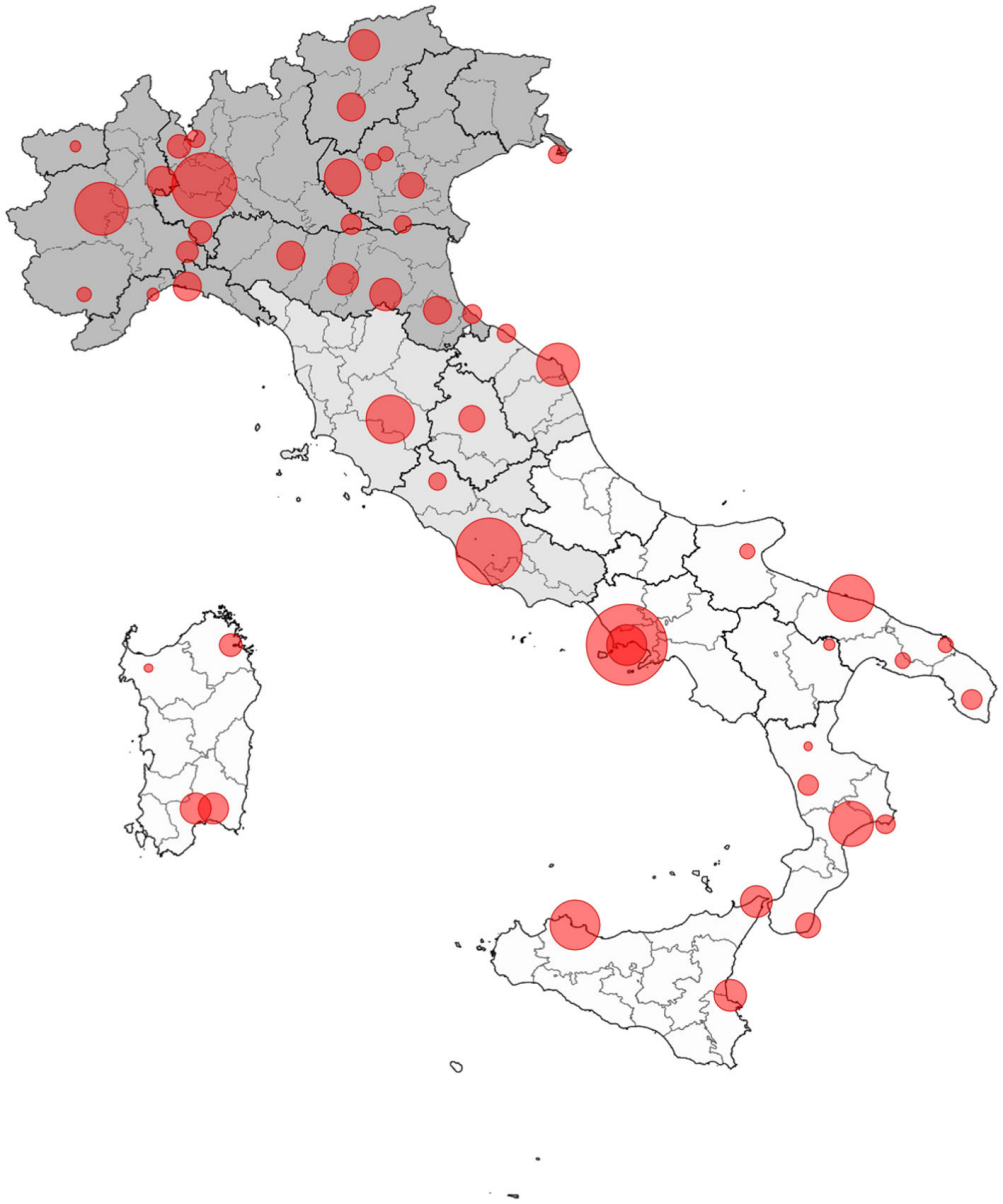
Geographical distribution of centers participating in the study on DKA 2014–2016. Each bubble dimension represents the number of cases recruited by each center, ranging from 5 to 379 (North Italy in dark gray, Center Italy in light gray, South Italy in white).

Fifty-four of the fifty-eight centers (93%) participated in this study (Figure 1). Each center prospectively registers data on all children with T1D at the time of diagnosis. This process follows agreed upon procedures established in 1997 by the Italian Insulin-Dependent Diabetes Registry (RIDI) (14), that are based on a standardized case report format. This study included all new cases of T1D over 0.5 and under 15 years of age presenting between January 1st, 2014 and December 31st, 2016. The diagnosis was confirmed with the presence of at least one of the antibodies against beta-cell antigens, islet cell antibodies (ICA), insulin antibodies (IA), glutamate dehydroxilase antibodies (GADA), islet antigen 2 antibodies (IA2A), and zinc-transporter protein 8 (ZnT8A). All other forms of diabetes were excluded. As the participating centers prospectively record all new cases of T1D that occur every year, the completeness of data collected on diabetes cases in the study period was higher than 95%. Moreover, detailed information on parents' socioeconomic status was mandatory for this study, while such information was not available for the analysis of the previous period (2004–2013).

### Variables

Data collected consisted on date of birth, date of diabetes diagnosis, gender, region of residence at diagnosis, education, and occupation levels of single or both parents, ethnic minority status, first-degree relative(s) with T1D, venous pH, bicarbonate levels. Data were anonymously sent to the coordinating center between January 1st and May 31st, 2017 and a unique database was obtained for the analysis.

The International Society for Pediatric and Adolescent Diabetes (ISPAD) criteria for DKA was used as a standard reference (15) (overall: pH < 7.30 or serum bicarbonate <15 mmol/L; severe: pH < 7.1, serum bicarbonate <5 mmol/L). Age at diagnosis was grouped in 0.5–4, 5–9, and 10–14 years; residence at diagnosis was classified as North, Center, and South (including Sicily and Sardinia); ethnic minority status was defined as at least one parent born outside the country with a positive migration history, or using a list of ethnic categories (African, Asian, mixed). Parents' education level was classified as low (without a high school diploma: <13 schooling years), and high (high school diploma attainment or university studies: at least 13 schooling years). Parents' occupation was collected following the classification of the Italian National Institute of Statistics, which is totally cross-linkable with the International Standard Classification of Occupations. They are grouped in two levels: low (unoccupied, unskilled and semi-skilled workers, manual workers and craftsmen), and high (legislators, senior officials and managers, professionals, technicians and associate professionals, sales workers, small business and farm owners, administrators and higher executives).

All procedures were conducted in accordance with the ethical standards of each participating center. Parents' written informed consent was collected by each participating center. No ethics committee approval was required, as this study was based on data routinely recorded in clinical practice and anonymously transmitted to the coordinating center.

### Statistical Methods

This analysis included two categories of DKA, overall (pH < 7.3) and severe (pH < 7.1). They were estimated as percent frequency and 95% confidence interval (95% CI) based on the binomial distribution and evaluated by gender, age group, geographical area of residence at diagnosis, parents' level of education and occupation, family history of type 1 diabetes, and ethnic minority status. The Chi-square test was used to compare the two groups. Diabetic ketoacidosis frequency between the two time periods were compared overall and by age groups using z-test for two proportions. The Kolmogorov-Smirnov test was used to compare the distributions of pH median values for a single year of age between the two time periods.

Two multiple logistic regression models were used to evaluate factors associated with DKA frequency. The dependent variable was DKA, considered as present vs. absent in the first model and as severe vs. absent in the second one. Gender, age class, geographic area of residence (Central Italy was the reference category), father and mother education and occupation level, and ethnic minority status were used as independent variables. Due to missing data for parents' level of education and occupation, a third category was added that included these missing values. The Likelihood Ratio test was used to detect the variables that were included in each model. The model's goodness of fit was evaluated by the Hosmer-Lemeshow test. All estimates were evaluated using the 95% CI.

All the statistical analyses were performed using the R.3.1.2 statistical package; a probability lower than 0.05 was used to assess statistical significance.

## Results

There were 2,808 newly diagnosed cases of T1D during the study period, of whom 2,679 (95.4%) were evaluable for DKA frequency. A total of 989 children had DKA at diabetes presentation (36.9, 95% CI: 35.1–38.8); 321 had severe DKA (12.0, 95% CI: 10.8–13.3).

### Comparison of DKA Frequency Between 2014–2016 and 2004–2013

**Figure 2:**
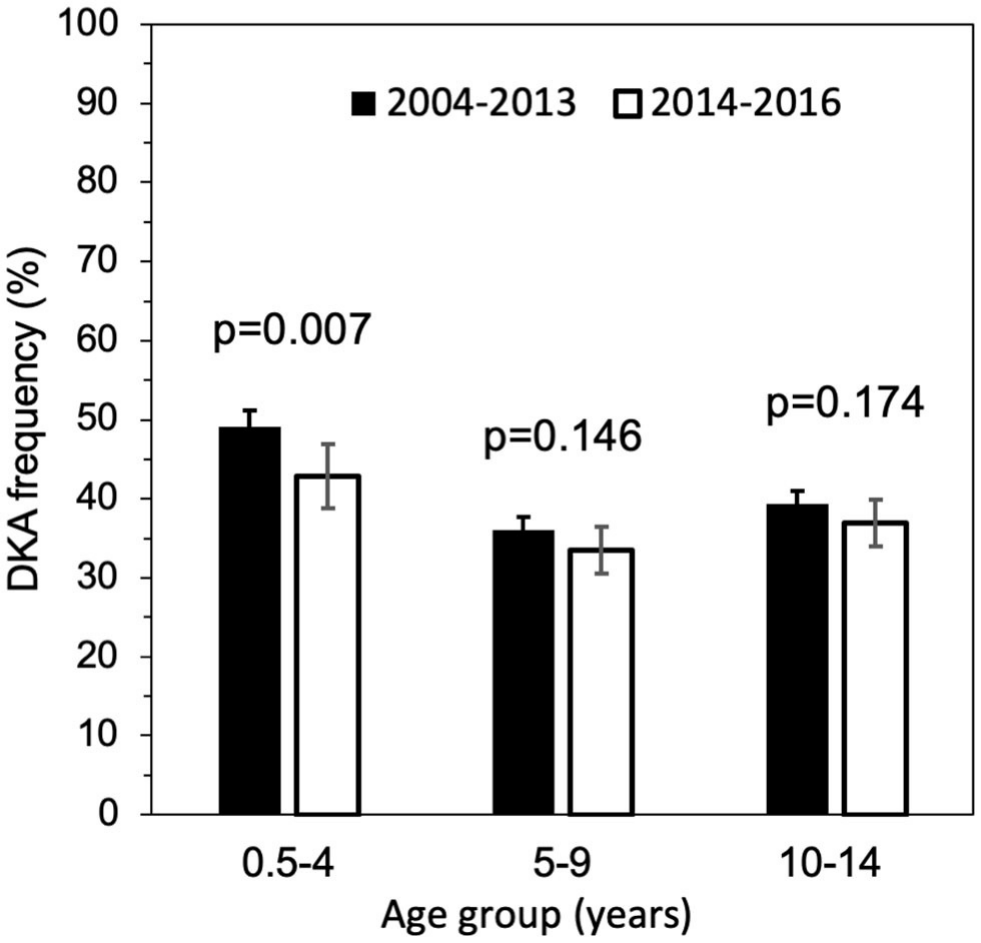
DKA frequency and 95% CI by age groups, comparison between 2004–2013 and 2014–2016.

**Figure 3:**
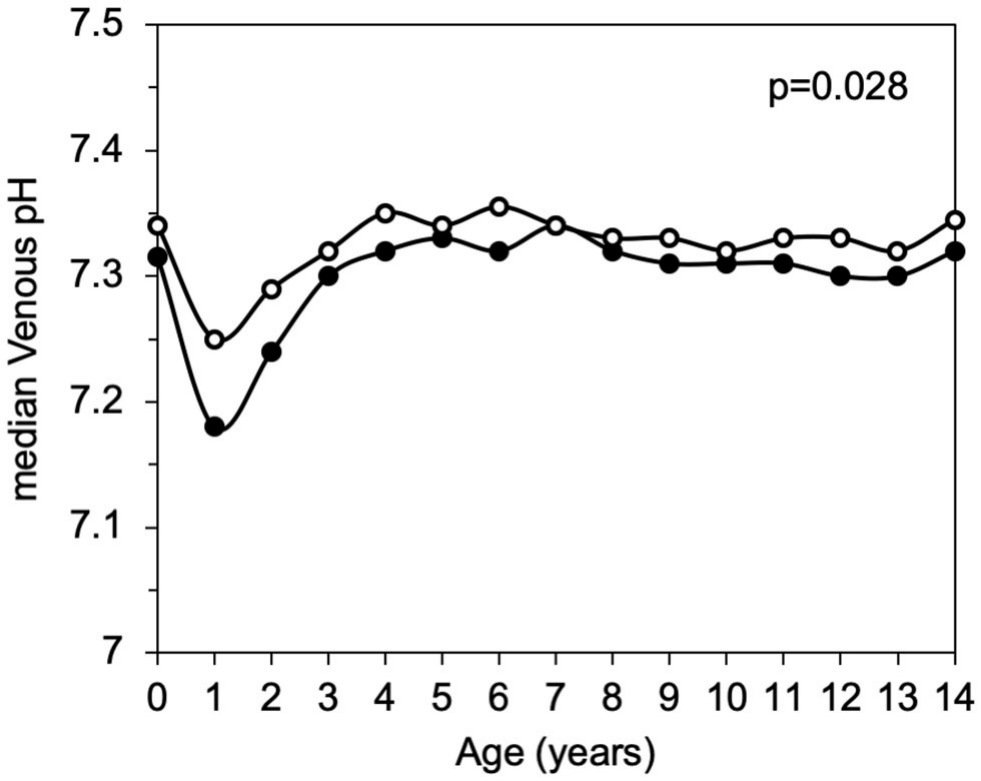
Median pH values by age, comparison between 2004–2013 (black dots) and 2014–2016 (white dots).

DKA frequency was significantly lower in 2014–2016 than 2004–2013 (40.3, 95% CI: 39.3–41.4, *p* = 0.002). Figure 2 shows a significant reduction of DKA frequency in children with 0.5–4 years diagnosed during 2014–2016 compared to 2004–2013 period (*p* = 0.007). Figure 3 shows that median venous pH values at diabetes presentation were significantly higher in 2014–2016 compared to the 2004–2013 period (*p* = 0.028).

### Socioeconomic Factors

Significantly higher DKA frequencies (both overall and severe) were found in children of 0.5–4 years of age, with both a low level of mother's education and parents' occupation (Table 1). The frequency of severe DKA was significantly higher in children when the father had a low level of education. No significant difference was found according to gender, residence at diagnosis, or minority status.

**Table 1:** Cases of Type 1 diabetes, DKA frequency at diagnosis and 95% confidence interval according to characteristics of children and parents between 2014 and 2016.

		**Type 1** **diabetes cases**		**Overall DKA** **(pH < 7.3)**			**Severe DKA** **(pH < 7.1)**	
			**Cases**	**Frequency (95% CI)**	***p***	**Cases**	**Frequency (95%CI)**	***p***
	Overall	2,679	989	36.9 (35.1–38.8)		321	12.0 (10.8–13.3)	
Gender	Female	1,261	484	38.4 (35.7–41.1)	0.149	161	12.8 (11–14.7)	0.289
	Male	1,418	505	35.6 (33.1–38.2)		160	11.3 (9.7–13)	
Age class	0.5–4 years	605	259	42.8 (38.8–46.9)	<0.001	106	17.5 (14.6–20.8)	<0.001
	5–9 years	1,032	346	33.5 (30.6–36.5)		92	8.9 (7.2–10.8)	
	10–14 years	1,040	384	36.9 (34.0–39.9)		123	11.8 (9.9–13.9)	
Residence at diagnosis	North	1,112	416	37.4 (34.6–40.3)	0.426	129	11.6 (9.8–13.6)	0.476
	Center	506	196	38.7 (34.5–43.1)		60	11.9 (9.2–15.0)	
	South	1,061	377	35.5 (32.6–38.5)		132	12.4 (10.5–14.6)	
Father education	Low	593	216	36.4 (32.5–40.4)	0.357	86	14.5 (11.8–17.6)	0.032
	High	785	266	33.9 (30.6–37.3)		78	9.9 (7.9–12.2)	
Mother education	Low	517	209	40.4 (36.2–44.8)	0.001	89	17.2 (14.1–20.8)	<0.001
	High	867	276	31.8 (28.7–35.1)		77	8.9 (7.1–11)	
Father occupation level	Low	719	288	40.1 (36.5–43.7)	<0.001	108	15.0 (12.5–17.8)	<0.001
	High	811	254	31.3 (28.1–34.6)		76	9.4 (7.5–11.6)	
Mother occupation level	Low	758	301	39.7 (27.7–43.3)	0.001	121	16 (13.4–18.8)	<0.001
	High	773	239	30.9 (27.7–34.3)		62	8.0 (6.2–10.2)	
Family history of type 1 diabetes	No	2,334	912	39.1 (37.1–41.1)	<0.001	298	12.8 (11.4–14.2)	<0.001
	Yes	212	41	19.3 (14.3–25.3)		10	4.7 (2.3–8.5)	
Ethnic Minority Status	No	1,830	690	37.7 (35.5–40.0)	0.058	221	12.1 (10.6–13.7)	0.123
	Yes	457	195	42.7 (38.1–47.3)		58	12.7 (9.8–16.1)	

*p-values refer to Chi-squared test*.

Table 2 shows factors associated with overall and severe DKA. The youngest children had a higher probability of overall and severe DKA at T1D diagnosis. The mother's high level of education, mother's or father's high level of occupation and the presence of at least one first-degree member with T1D decreased the probability of having DKA at diagnosis. Children living in Southern Italy had a higher probability of diagnosis with severe DKA than children living in Central Italy.

**Table 2:** Association between gender, age class, residence, parents' level of education, family history of type 1 diabetes, minority status and diabetic ketoacidosis at diagnosis of Type 1 Diabetes.

		**pH** **<** **7.3 vs. pH** **≥** **7.3**			**pH** **<** **7.1 vs. pH** **≥** **7.3**		
**Factors**		**OR**	**95% CI**	***p***	**OR**	**95% CI**	***p***
Gender	M vs. F	0.86	0.72–1.02	0.091	0.83	0.63–1.09	0.172
Age class	5–9 vs. 0.5–4	0.64	0.51–0.81	<0.001	0.41	0.29–0.59	<0.001
	10–14 vs. 0.5–4	0.76	0.61–0.96	0.019	0.65	0.47–0.9	0.009
Residence at diagnosis	North vs. Center	1.00	0.77–1.3	0.993	1.14	0.76–1.74	0.527
	South vs. Center	1.19	0.91–1.57	0.211	1.60	1.05–2.46	0.031
Level of Education	Father, High vs. Low	1.29	0.96–1.73	0.088	1.26	0.8–1.97	0.318
	Father, Missing vs. Low	1.00	0.41–2.44	0.999	2.02	0.54–7.03	0.278
	Mother, High vs. Low	0.69	0.51–0.93	0.014	0.52	0.33–0.81	0.004
	Mother, Missing vs. Low	0.92	0.38–2.21	0.851	0.39	0.11–1.44	0.146
Level of Occupation	Father, High vs. Low	0.72	0.55–0.94	0.018	0.77	0.51–1.16	0.215
	Father, Missing vs. Low	1.26	0.78–2.06	0.344	1.16	0.55–2.5	0.708
	Mother, High vs. Low	0.76	0.58–0.99	0.045	0.53	0.34–0.81	0.004
	Mother, Missing vs. Low	0.70	0.43–1.12	0.137	0.56	0.27–1.14	0.116
Family history of type 1 diabetes	Yes vs. No	0.39	0.27–0.57	<0.001	0.29	0.14–0.56	0.001
Ethnic Minority Status	Yes vs. No	1.23	0.98–1.53	0.075	1.03	0.72–1.45	0.873

Results of multiple logistic regression analyses.

First model (pH < 7.3 vs. pH ≥ 7.3): Hosmer-Lemeshow test: χ^2^ = 8.922, df = 8, p-value 0.349; Likelihood Ratio test: χ^2^ = 88, df =15, p < 0.001.

*Second model (pH < 7.1 vs. pH ≥ 7.3): Hosmer-Lemeshow test: χ^2^ = 12.25, df = 8, p-value 0.140; Likelihood Ratio test: χ^2^ = 88.6, df = 15, p < 0.001*.

## Discussion

This national study of 2,679 children with type 1 diabetes under the age of 15 demonstrated that a lower SES is associated with an increased probability of having ketoacidosis at the diagnosis of T1D between 2014 and 2016. Both parents' educational and occupational dimensions were related to the probability of DKA at diagnosis. However, the mother's SES appears to play a more important role compared to the father, the mothers' low level of education and occupation increased the risk of DKA at the diagnosis of diabetes. Ethnic minority status was not found to be significantly associated with DKA frequency. Furthermore, the probability of having DKA was still high in Italy during the 3 year period 2014–2016, even if we found a reduction in the percentage of children with ketoacidosis compared to the 10 year period from 2004–2013. These results suggest a delayed diagnosis in too many children. The huge difference in the risk of overall DKA previously reported between Central and Southern Italy (6) was not confirmed in this study, however the frequency of severe DKA was still much higher in the South compared to Central Italy.

### Parental Education and Occupational Levels

Few studies have analyzed the association between parental educational level and DKA at the diagnosis of diabetes, suggesting that education affects the chance of developing ketoacidosis (15, 16). In addition, the level of family income (17) and socio-economic status (17) can be a risk factor for ketoacidosis. Moreover, results from completed studies are not consistent. A low level of maternal education was associated with a higher risk of DKA in 401 children under 15 years of age in Lithuania (18) while in a series of 474 children under 17 years of age in Poland an association between delayed diagnosis of type 1 diabetes and level of maternal education (19) was not found. Likewise, in a study conducted in Korea, no association was found between the level of parental education and the risk of DKA in a group of 361 children, however, the children of parents with low education showed an increased risk of severe DKA (20).

In this study, the high level of maternal education has shown to be highly protective for both overall and severe DKA. It is possible that mothers, who provide more direct care of children than fathers in Italy, may notice diabetes symptoms earlier if they have a high level of education. In this study the father's educational level was not associated with the DKA at diagnosis of diabetes.

However, we observed that the father's high occupation level had a protective role toward overall DKA. The mother's occupation plays an important role in the prevention of both overall and severe DKA. It is plausible that a high level of occupation allows parents to have more exchanges with other people, and live in a favorable social network to obtain useful information that can lead to an early detection of the characteristic symptoms of diabetes.

### Ethnic Minority Status

A 2006–2016 study (7), demonstrated a significantly higher percentage of children from ethnic minority groups who presented with DKA at the diagnosis of T1D than peers from non-ethnic minority groups in Italy. This same result was not found in the case series analyzed during 2014–2016. This is in contrast with results from other studies in which children belonging to minority ethnic groups showed higher frequency of DKA (21). It is likely that the inclusion of parents' level of education and occupation in the multiple logistic regression analysis reduced the independent effect of ethnic minority status. Indeed, the access to the Italian healthcare system, that warrants services, medications, and devices free of charge, allows all residents to receive assistance by avoiding differences based on ethnic minority.

### Younger Children

Other studies have reported a greater probability for DKA at diagnosis in younger children (7, 12, 22, 23). Our study confirmed that younger children had a higher probability of DKA than school age children and adolescents. However, when comparing these results with data reported over the past 10 years, we have observed a significant reduction in the percentage of DKA in the age group 0.5–4 years. Although this result is encouraging, it highlights that the probability is still too high and the presence of diabetes symptoms in younger children requires more attention from parents and pediatricians.

### Awareness Campaigns

A few studies have highlighted the effectiveness of awareness campaigns to prevent DKA at diagnosis of diabetes during last 20 years (8, 10, 24–26). To date, due to methodological limitations of those studies, it is not possible to make a definitive conclusion on their effectiveness (8). To support the findings from the literature, it is difficult to associate the reduction of DKA frequency we have observed in this study and the implementation of the national awareness campaign promoted by ISPED. This is due to factors such as the lack of structured educational interventions, i.e., meetings with pediatricians were not simultaneously and systematically organized, variation in implementation by pediatric centers, i.e., some pediatric centers of the country started their own campaign earlier than others, and most importantly that the awareness campaign is an ongoing activity. To determine the effectiveness of this intervention in Italy, a large number of cases over a longer period of time need to be evaluated. In addition, several other non-structured and less conventional initiatives intended to prevent DKA at diagnosis were carried out during 2014 and 2015, such as messages by association of parents of children with diabetes launched through social media or sportive events, i.e., periodical marathons across Italy (http://www.weloveinsulina.it).

These initiatives could have contributed to the observed reduction of DKA and demonstrate the importance of non-conventional communication systems to reinforce efforts aimed to spread education on DKA prevention to both the general population and health care providers. However, it should be also noted that socioeconomic status and relation to social media is controversial; lower socioeconomic status might be related to an increased use of unproductive social media use and abuse. Beyond the reports of cumulative effects of various actions, the results from this study also demonstrate the need to explore education and awareness-raising campaigns tailored to the needs of families, in particular for those with a low level of parent education and the ability to access resources and for children that are a younger age. Ideally, the frequency of DKA should be reduced to a minimum level comparable to that found in children with islet-positive autoantibodies detected by the Bavarian screening program (27), who developed overt disease. The proportion of children reporting pH value <7.30 at type 1 diabetes diagnosis was <5%.

### Strengths and Limitations

The Network of ISPED for DKA Study and Prevention involved 54 out of 58 Italian centers for pediatric diabetes. Strengths of this study included nationwide collaboration, which allowed the collection of a large number of cases of T1D at diagnosis under the age of 15 years. This collaboration has lasted from more than 30 years and is based on the Network of ISPED for DKA Study and Prevention of a national scientific society, involving pediatric diabetologists skilled on research methodology. In addition, data recording and collection was based on standard criteria, and used consistent definitions of DKA over time.

Limitations of the study included missing data for some crucial information. We included only cases of type 1 diabetes with information on DKA status; nevertheless, cases without information on pH and/or bicarbonate were <5% and they might not have negatively affected DKA frequency estimation. A high percentage of missing data was found in socioeconomic variables, and there was a consistent distribution of missing values for parents. To overcome this limitation and minimize the loss of information in the statistical analysis, we included a category for missing values both in parents' education and occupation.

## Conclusion

This study demonstrated a decrease in the presence of DKA among younger children in the 2014–2016 period. However, even though this reduction is clinically relevant, there is still an unacceptably high frequency of DKA in the diagnosis of children with T1D under the age of 15. Socioeconomic inequalities, namely low education and occupational levels, were associated with the increased probability of DKA at diagnosis of T1D. Given the results of this study, areas of further investigation consist of undertaking further research to explore barriers to early diagnosis of T1D among children under 15 in Italy especially in families that experience greater socioeconomic inequalities. Undertaking such studies may provide information on both the structural barriers and the educational needs of this distinct population. Based on this information, future interventions aimed at improving the health outcomes of children living with T1D in Italy might be more effective.

## Data Availability Statement

The data analyzed in this study is subject to the following licenses/restrictions: Datasets consist of data routinely recorded in clinical practice and anonymously transmitted to the coordinating center. Requests to access these datasets should be directed to Valentino Cherubini, valentino.cherubini@gmail.com.

## Ethics Statement

All procedures were conducted in accordance with the ethical standards of each participating center. Parents' written informed consent was collected by each participating center. No ethics committee approval was required, as this study was based on data routinely recorded in clinical practice and anonymously transmitted to the coordinating center.

## Author Contributions

VC and RG designed the study and wrote the manuscript. RG carried out the statistical analysis. CM, RBon, FCa, FCi, GD'A, AF, DI, AI, FL, GM, IP, EP, BP, IR, CR ST, and RS researched data and contributed to review the manuscript. VC is the guarantor of this work and, as such, had full access to all the analyzed data in the study. RG takes the responsibility for the integrity of the data and accuracy of the data analysis. RBow contributed to the discussion, peer-reviewed, and supported revisions of the paper. All data owners have given permission for publication. All authors revised and approved the final version of the manuscript and are accountable for all aspects of the work.

## Network of the Italian Society of Pediatric Endocrinology and Diabetes (ISPED) for DKA Study and Prevention

Riccardo Lera, Alessandria, rlera@ospedale.al.it; Monica Marino, Ancona, monicamarino96@icloud.com; Valentina Tiberi, Ancona, v.tiberi86@gmail.com; Adriana Bobbio, Aosta, adriana.bobbio@gmail.com; Paolo Serravalle, Aosta, pserravalle@ausl.vda.it; Eleonardo Schieven, Arzignano, eleonardoschieven@gmail.com; Maurizio Delvecchio, Bari, mdelvecchio75@gmail.com; Federica Ortolani, Bari, federicaortolani@hotmail.com; Stefano Zucchini, Bologna, stefano.zucchini@aosp.bo.it; Petra Reinstadler, Bolzano, petra.reinstadler@sabes.it; Ylenia Girtler, Bolzano, ylenia.girtler@sabes.it; Elena Prandi, Brescia, prandi.elena@gmail.com; Francesco Gallo, Brindisi, fragallo1966@gmail.com; Paola Frongia, Cagliari, annapaolafrongia@aob.it; Alfonso La Loggia, Caltanissetta, alfonso.laloggia@gmail.com; Giuliana Cardinale, Casarano, giulianacardinale@libero.it; Sergio Lucieri, Castrovillari, sergiolucieri@libero.it; Filomena Stamati, Castrovillari, filostamati@virgilio.it; Letizia Tomaselli, Catania, letitom56@hotmail.com; Sara Monti, Cesena, sara.monti@♀auslromagna.it; Maria Zampolli, Como, maria.zampolli@tin.it; Rosaria De Marco, Cosenza, dmcrosaria@libero.it; Andrea Scaramuzza, Cremona, a.scaramuzza@gmail.com; Nicola Lazzaro, Crotone, niclaz@libero.it; Valeria De Donno, Cuneo, dedonno.v@ospedale.cuneo.it; Lorenzo Lenzi, Firenze, lr.lenzi@meyer.it; Maria Susanna Coccioli, Francavilla, susanna.coccioli@gmail.com; Alberto La Valle, Genova, albertolavalle89@gmail.com; Nicola Minuto, Genova, nicolaminuto@gaslini.org; Mariella Bruzzese, Locri, mariellabruzzese@libero.it; Elena Mazzali, Mantova, elena.mazzali@asst-mantova.it; Silvia Sordelli, Mantova, silviasordelli2@gmail.com; Antonina Tirendi, Mantova, antonina.tirendi@asst-mantova.it; Giuseppina Salzano, Messina, gsalzano@unime.it; Raffaella Ditonno, Milano, ditonno.raffaella@gmail.com; Valeria Favalli, Milano, favalli.valeria@gmail.com; Giulio Frontino, Milano, frontino.giulio@gmail.com; Lorenzo Iughetti, Modena, iughetti.lorenzo@unimre.it; Patrizia Bruzzi, Modena, bruzzi.patrizia@aou.mo.it; Andrea Rigamonti, Milano, rigamonti.andrea@hsr.it; Alberto Casertano, Naples, casertanoalberto@gmail.com; Enza Mozzillo, Naples, mozzilloenza@gmail.com; Alessia Piscopo, Naples, alessia.piscopo@hotmail.com; Angela Zanfardino, Naples, angela.zanfardino@unicampania.it; Erica Pozzi, Novara, ericapozzi@yahoo.it; Silvia Savastio, Novara, savastio.silvia@gmail.com; Gavina Piredda, Olbia, gav.pi@libero.it; Carlo Moretti, Padova, carlo.moretti@aopd.veneto.it; Rosalia Roppolo, Palermo, roppoloros@gmail.com; Brunella Iovane, Parma, biovane@ao.pr.it; Valeria Calcaterra, Pavia, v.calcaterra@smatteo.pv.it; Maria Giulia Berioli, Perugia, mgiuliaberioli@gmail.com; Martina Biagioni, Pesaro, biagioni.martina@libero.it; Vanna Graziani, Ravenna, vanna.graziani@gmail.com; Tosca Suprani, Ravenna, tosca.suprani@auslromagna.it; Annalisa Pedini, Rimini, annalisa.pedini@auslromagna.it; Stefania Innaturato, Rovigo, stefania.innaturato@libero.it; Irene Rutigliano, San Giovanni Rotondo, irene.rutigliano@libero.it; Michele Sacco, San Giovanni Rotondo, m.sacco@operapadrepio.it; Gianfranco Meloni, Sassari, swati@hns.net.in; Graziella Fichera, Savona, g.fichera@asl2.liguria.it; Alberto Gaiero, Savona, a.gaiero@asl2.liguria.it; Luisa De Sanctis, Torino, luisa.desanctis@unito.it; Davide Tinti, Torino, davide.tinti@unito.it; Michela Trada, Torino, m.trada72@gmail.com; Lucia Paola Guerraggio, Tradate, luciapaola.guerraggio@asst-settelaghi.it; Silvia Zonca, Tradate, silvia.zonca@asst-settelaghi.it; Vittoria Cauvin, Trento, vittoria.cauvin@apss.tn.it; Roberto Franceschi, Trento, robertofranceschi@yahoo.it; Elena Faleschini, Trieste, elena.faleschini@burlo.trieste.it; Gianluca Tornese, Trieste, gianluca.tornese@burlo.trieste.it; Marco Marigliano, Verona, marco.marigliano@univr.it; Claudia Arnaldi, Viterbo, c.arnaldi@inwind.it.

## Conflict of Interest

The authors declare that the research was conducted in the absence of any commercial or financial relationships that could be construed as a potential conflict of interest.
